# Perinatal depression and risk of maternal cardiovascular disease: a Swedish nationwide study

**DOI:** 10.1093/eurheartj/ehae170

**Published:** 2024-06-18

**Authors:** Donghao Lu, Unnur A Valdimarsdóttir, Dang Wei, Yufeng Chen, Ole A Andreassen, Fang Fang, Krisztina D László, Emma Bränn

**Affiliations:** Institute of Environmental Medicine, Unit of Integrative Epidemiology, Karolinska Institutet, Nobels väg 13, 17177 Stockholm, Sweden; Institute of Environmental Medicine, Unit of Integrative Epidemiology, Karolinska Institutet, Nobels väg 13, 17177 Stockholm, Sweden; Center of Public Health Sciences, Faculty of Medicine, University of Iceland, Reykjavík, Iceland; Institute of Environmental Medicine, Unit of Integrative Epidemiology, Karolinska Institutet, Nobels väg 13, 17177 Stockholm, Sweden; Institute of Environmental Medicine, Unit of Integrative Epidemiology, Karolinska Institutet, Nobels väg 13, 17177 Stockholm, Sweden; NORMENT Centre, Institute of Clinical Medicine, University of Oslo and Division of Mental Health and Addiction, Oslo University Hospital, Oslo, Norway; KG Jebsen Centre for Neurodevelopmental Disorders, University of Oslo, Oslo, Norway; Institute of Environmental Medicine, Unit of Integrative Epidemiology, Karolinska Institutet, Nobels väg 13, 17177 Stockholm, Sweden; Department of Global Public Health, Karolinska Institutet, Stockholm, Sweden; Department of Public Health and Caring Sciences, Uppsala University, Uppsala, Sweden; Institute of Environmental Medicine, Unit of Integrative Epidemiology, Karolinska Institutet, Nobels väg 13, 17177 Stockholm, Sweden

**Keywords:** Perinatal depression, Cardiovascular disease, Postpartum depression, Heart failure, Stroke, Ischaemic heart disease

## Abstract

**Background and Aims:**

Increasing evidence suggests that some reproductive factors/hazards are associated with a future risk of cardiovascular disease (CVD) in women. While major (non-perinatal) depression has consistently been associated with CVD, the long-term risk of CVD after perinatal depression (PND) is largely unknown.

**Methods:**

A nationwide population-based matched cohort study involving 55 539 women diagnosed with PND during 2001–14 in Sweden and 545 567 unaffected women individually matched on age and year of conception/delivery was conducted. All women were followed up to 2020. Perinatal depression and CVD were identified from Swedish national health registers. Using multivariable Cox models, hazard ratios (HR) of any and type-specific CVD according to PND were estimated.

**Results:**

The mean age at the PND diagnosis was 30.8 [standard deviation (SD) 5.6] years. During the follow-up of up to 20 years (mean 10.4, SD 3.6), 3533 (6.4%) women with PND (expected number 2077) and 20 202 (3.7%) unaffected women developed CVD. Compared with matched unaffected women, women with PND had a 36% higher risk of developing CVD [adjusted HR = 1.36, 95% confidence interval (CI): 1.31–1.42], while compared with their sisters, women with PND had a 20% higher risk of CVD (adjusted HR = 1.20, 95% CI 1.07–1.34). The results were most pronounced in women without a history of psychiatric disorder (*P* for interaction < .001). The association was observed for all CVD subtypes, with the highest HR in the case of hypertensive disease (HR = 1.50, 95% CI: 1.41–1.60), ischaemic heart disease (HR = 1.37, 95% CI: 1.13–1.65), and heart failure (HR 1.36, 95% CI: 1.06–1.74).

**Conclusions:**

Women with PND are at higher risk of CVD in middle adulthood. Reproductive history, including PND, should be considered in CVD risk assessments of women.

## Structured Graphical Abstract

**Structured Graphical Abstract:**
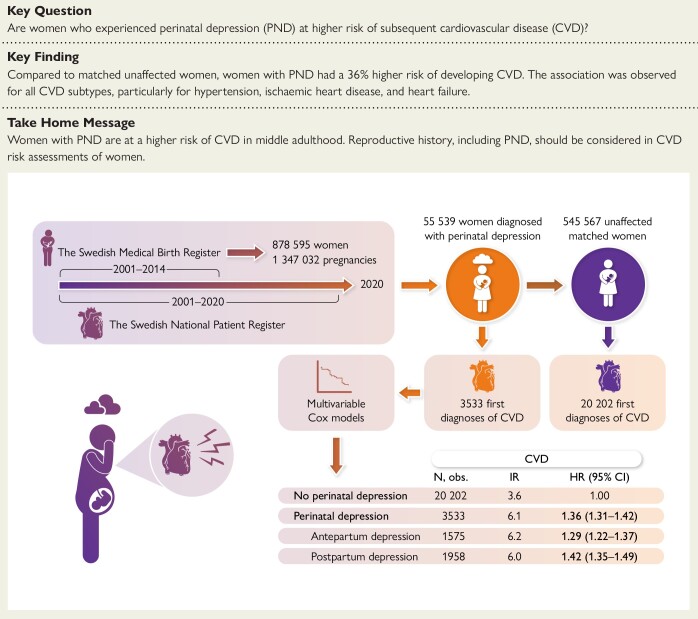
Overview of the study design and the main results: association between perinatal depression (PND) and any subsequent cardiovascular disease (CVD) as well as separate analyses for antepartum and postpartum depression. CI, confidence interval; HR, hazard ratio; IR, incidence rate per 1000 person-years; N, obs., number of CVD events.

**See the editorial comment for this article ‘Perinatal depression and incident maternal cardiovascular disease: a neglected association’, by A. Meaidi, https://doi.org/10.1093/eurheartj/ehae340.**

## Introduction

Historically, cardiovascular disease (CVD) research has mainly focused on male populations.^1^ It led to the identification of risk factors important for men, including hyperlipidaemia, obesity, diabetes, and smoking, although these factors are also linked to CVD risk in women.^2,3^ While research regarding women’s cardiovascular health has increased significantly during the past decades, female-specific risk factors for CVD remain understudied and largely unrecognized. Increasing evidence suggests that reproductive history, including miscarriage, pre-term birth, stillbirth, pre-eclampsia, and gestational diabetes, may be used to inform future risk of CVD in women.^4,5^

While pregnancy complications and outcomes often intertwine with perinatal mental health,^6^ the latter is seldomly assessed as part of reproductive history in clinical practice. Notably, many of these reproductive factors have been linked to perinatal depression (PND),^7–9^ a depressive episode occurring during or in the period after pregnancy. Moreover, previous studies have consistently shown a bidirectional association between non-PND and CVD.^10^ Potential mechanisms linking PND with CVD during pregnancy have been proposed,^11^ and antepartum depression (APD) has been associated with short-term risk of CVD.^12^ However, the association of PND with risk of subsequent CVD, particularly in the long term, is largely unknown.

Leveraging the nationwide register data in Sweden, in the present study, we aimed to characterize and assess the risk of CVD following PND using a population-based matched cohort.

## Methods

### Study design

The study was approved by the Swedish Ethics Review Authority (2016/288–31/1; 2018/1515-31; 2021/03315). Leveraging the Swedish Medical Birth Register (MBR),^13^ we identified 878 595 women with 1 347 032 pregnancies who gave birth during 2001–14 in Sweden. The MBR was established in 1973 and includes information on virtually all births in the country. Using the women’ personal identification number, data from MBR were linked to the Swedish National Patient Register (NPR) and to the Swedish Prescribed Drug Register (PDR). The NPR was established in 1964 including data on inpatient care and from 2001 also includes data on specialized outpatient care. By 1981, the NPR covered >80% of the inpatient care in Sweden and our data start from 1969. The PDR was established in July 2005 and includes all prescribed drugs dispensed at pharmacies in Sweden. With further record linkage to the Cause of Death Register and the Total Population Register, we identified deaths and emigration during the follow-up; data on emigration were available to us until 2014. After excluding 42 009 twin pregnancies, 18 433 pregnancies after the pregnancy with PND, and 212 erroneous records, the study sample consisted of 1 286 378 pregnancies from 867 945 women.

In order to investigate the association between PND and the subsequent risk of CVD, we conducted a matched cohort study based on the study sample. Briefly, we identified all women with a first-ever PND, defined as a depression diagnosis or prescription of antidepressant (available from July 2005 in the PDR) recorded from the estimated date of conception to 1 year postpartum.^14–17^ We retrieved information on year and month of delivery and estimated gestational length (generally estimated using from routine ultrasound around gestational week 18) from MBR to calculate the expected conception date. Using incidence density sampling, for each woman with APD, we randomly sampled 10 women who were free of PND at the same gestational age, whereas, for each woman with postpartum depression (PPD), we randomly selected 10 women who were free of PND at the same postpartum day, from the entire study sample, whenever possible. The index woman with PND and the unaffected women were individually matched on age at delivery and calendar year of conception/delivery, resulting in 57 383 women with PND and 573 823 unaffected women. Date of PND diagnosis was used as the index date for both PND woman and their matched unaffected women.

**Figure 1:**
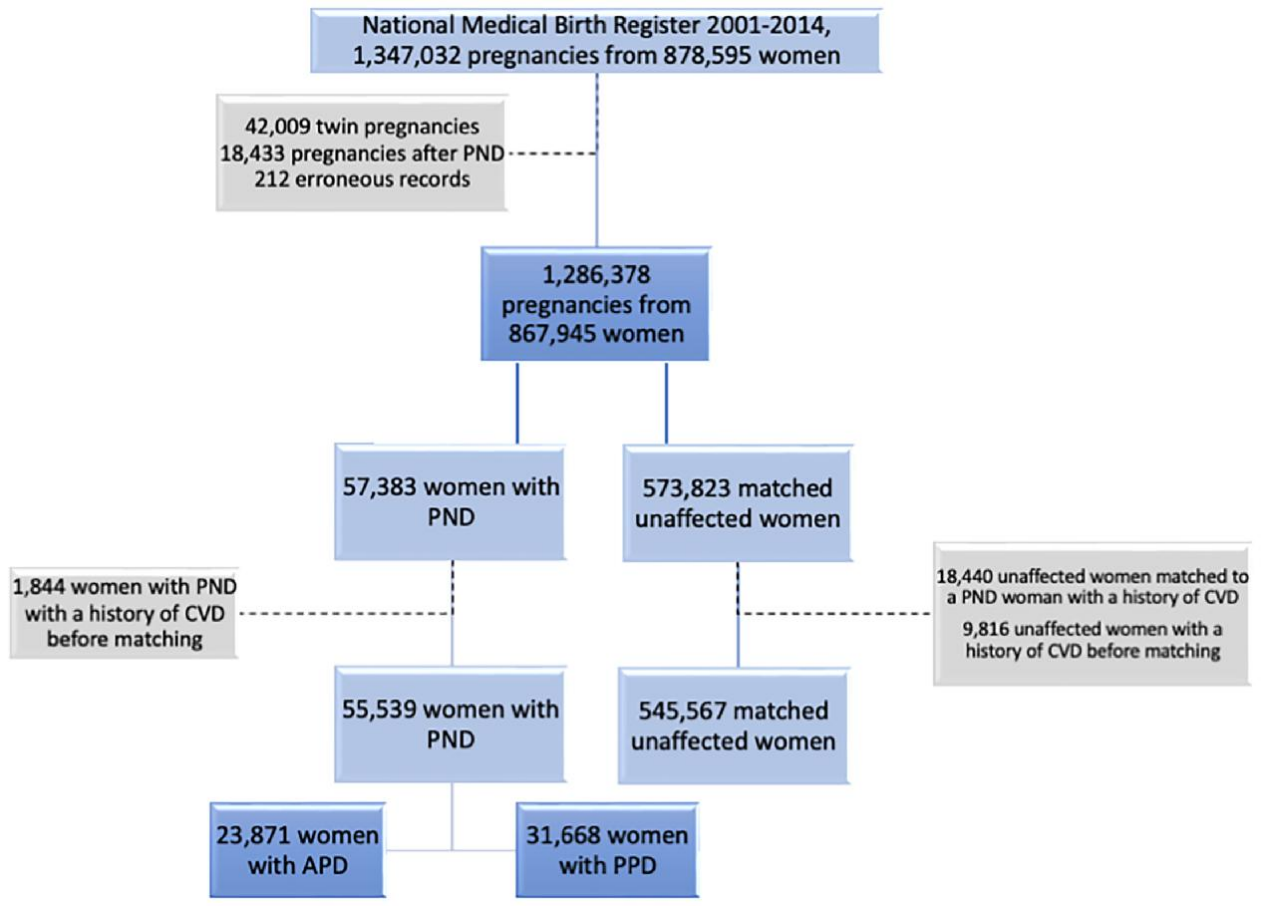
Flowchart of inclusion and exclusion criteria for establishing the study base. PND, perinatal depression; APD, antepartum depression; PPD, postpartum depression; CVD, cardiovascular disease

After the matching, we excluded women with a history of CVD before the index date, leaving 55 539 women with PND (23 871 APD and 31 668 PPD) and 545 567 matched unaffected women (*Figure 1*) for analysis. We then followed both PND women and unaffected women from the index date until diagnosis of CVD, emigration, death, 31 December 2020, or diagnosis of PND (only relevant for unaffected women), whichever came first.

### Ascertainment of perinatal depression

We defined PND as a depression diagnosis (see Supplementary data online, *Table S1*) recorded in the NPR or MBR or as a filled prescription of antidepressants (N06A) registered in the PDR (available from 2005). The date of diagnosis or prescription was not available when PND was ascertained only through the MBR. Consequently, the individual median date of pregnancy was assigned as date of diagnosis for women whose PND was identified only through the MBR. As different subtypes of PND have been characterized based on symptom onset,^18,19^ PND was further sub-divided into (i) APD if the first PND diagnosis/prescription was recorded during pregnancy and (ii) PPD if the first PND was recorded during the 12 months after delivery.

### Ascertainment of cardiovascular disease

In accordance with previous studies,^20,21^ we identified all CVD diagnoses (primary or secondary) registered in the NPR or death due to CVD as the underlying cause recorded in the Cause of Death Register during follow-up (see Supplementary data online, *Table S1*). Although we studied any CVD as the primary outcome, we also retrieved first-ever diagnoses of seven major CVD groups, including hypertensive disease, ischaemic heart disease, cerebrovascular disease, emboli and thrombosis, heart failure, arrhythmias, and other CVDs, respectively. Generally, the diagnosis of CVD has a good accuracy in NPR.^22^

### Covariates

Demographic variables included country of birth and marital status from the Total Population Register, years of education from the Education Register, and personal income from the Income and Taxation Register. Potential confounders, including smoking 3 months before pregnancy, body mass index (BMI) in early pregnancy, parity, gestational and pre-gestational diabetes, and pre-eclampsia, were identified from the NPR and MBR. As a potential association between PND and CVD could be attributed to other psychiatric disorders, we identified any psychiatric diagnosis before the index date from the NPR and classified them into depression and other psychiatric disorders. Adverse pregnancy outcomes may be used to inform future risk of CVD^4,5^ in women and have been associated with higher risks of PND^23,24^; therefore, the risk of CVD might be different between PND women with or without adverse pregnancy outcomes. We identified therefore adverse pregnancy outcomes from MBR, including pre-term delivery (before 37 weeks of gestation), caesarean section, congenital malformation in the offspring, and loss of offspring. Categories of all covariates are presented in *Table 1*. Missing data were coded as ‘unknown’ category for adjustment. The International Classification of Diseases (ICD) codes used to retrieve each medical condition are shown in Supplementary data online, *Table S1*.

**Table 1:** Characteristics of women with and without perinatal depression, *n* (%)

		Population		Sibling	
		No PND	PND	No PND	PND
Total *N*		545 567	55 539	16 420	13 804
Follow-up, years	Mean (SD)	10.3 (3.7)	10.4 (3.5)	11.5 (5.1)	10.6 (3.4)
Country of birth	Sweden	416 427 (76.3)	46 690 (84.1)	15 155 (92.3)	12 937 (93.7)
	Other	129 014 (23.6)	8836 (15.9)	1264 (7.7)	865 (6.3)
	Unknown	126 (0.0)	13 (0.0)	1 (0.0)	2 (0.0)
**At matching**					
Age, years	Mean (SD)	30.8 (5.6)	30.8 (5.6)	29.6 (5.1)	30.7 (5.1)
	<20	8416 (1.5)	863 (1.6)	314 (1.9)	143 (1.0)
	20–24	70 772 (13.0)	7148 (12.9)	2496 (15.2)	1552 (11.2)
	25–29	146 174 (26.8)	14 785 (26.6)	5241 (31.9)	3922 (28.4)
	30–34	177 192 (32.5)	17 981 (32.4)	5562 (33.9)	4927 (35.7)
	≥35	143 013 (26.2)	14 762 (26.6)	2805 (17.1)	3260 (23.6)
Calendar year	2001–04	26 990 (4.9)	2436 (4.4)	4239 (25.8)	538 (3.9)
	2005–09	233 019 (42.7)	23 940 (43.1)	5828 (35.5)	6173 (44.7)
	2010–14	285 558 (52.3)	29 163 (52.5)	6353 (38.7)	7093 (51.4)
Marital status	Married	215 029 (39.4)	26 692 (48.1)	8039 (49.0)	6636 (48.1)
	Not married	139 977 (25.7)	12 542 (22.6)	3888 (23.7)	2905 (21.0)
	Unknown	190 561 (34.9)	16 305 (29.4)	4493 (27.4)	4263 (30.9)
Education, years	≤ 9	61 094 (11.2)	9153 (16.5)	2130 (13.0)	2007 (14.5)
	10–11	52 011 (9.5)	7148 (12.9)	1872 (11.4)	1598 (11.6)
	12	155 498 (28.5)	16 067 (28.9)	5342 (32.5)	4243 (30.7)
	13–14	67 342 (12.3)	6683 (12.0)	1865 (11.4)	1641 (11.9)
	≥15	196 010 (35.9)	15 748 (28.4)	5109 (31.1)	4252 (30.8)
	Unknown	13 612 (2.5)	740 (1.3)	102 (0.6)	63 (0.5)
Income^a^	Q1	136 697 (25.1)	13 515 (24.3)	4366 (26.6)	3188 (23.1)
	Q2	134 698 (24.7)	15 771 (28.4)	4219 (25.7)	3342 (24.2)
	Q3	135 724 (24.9)	14 487 (26.1)	3944 (24.0)	3610 (26.2)
	Q4	138 448 (25.4)	11 766 (21.2)	3890 (23.7)	3664 (26.5)
**Pregnancy characteristics**					
Smoking 3 months before pregnancy	No	437 925 (80.3)	37 885 (68.2)	11 892 (72.4)	9540 (69.1)
	1–9 cig./day	41 835 (7.7)	6103 (11.0)	1732 (10.5)	1466 (10.6)
	≥10 cig./day	41 458 (7.6)	9057 (16.3)	2072 (12.6)	2153 (15.6)
	Unknown	24 349 (4.5)	2494 (4.5)	724 (4.4)	645 (4.7)
BMI in early pregnancy, kg/m^2^	<18.5	12 138 (2.2)	1297 (2.3)	373 (2.3)	319 (2.3)
	18.5–24.9	301 828 (55.3)	27 841 (50.1)	9010 (54.9)	7139 (51.7)
	25–29.9	127 715 (23.4)	13 425 (24.2)	3744 (22.8)	3244 (23.5)
	≥30	60 277 (11.0)	8234 (14.8)	1831 (11.2)	1956 (14.2)
	Unknown	43 609 (8.0)	4742 (8.5)	1462 (8.9)	1146 (8.3)
Parity	1	248 087 (45.5)	27 678 (49.8)	8479 (51.6)	6729 (48.7)
	2–3	267 038 (48.9)	24 362 (43.9)	7206 (43.9)	6264 (45.4)
	≥4	30 442 (5.6)	3499 (6.3)	735 (4.5)	811 (5.9)
Diabetes	No	535 954 (98.2)	54 119 (97.4)	16 177 (98.5)	13 488 (97.7)
	Gestational	6155 (1.1)	824 (1.5)	145 (0.9)	181 (1.3)
	Pre-gestational	3458 (0.6)	596 (1.1)	98 (0.6)	135 (1.0)
Pre-eclampsia	No	528 518 (96.9)	53 199 (95.8)	15 894 (96.8)	13 252 (96.0)
	Yes	17 049 (3.1)	2340 (4.2)	526 (3.2)	552 (4.0)
History of psychiatric disorder	No	495 967 (90.9)	31 513 (56.7)	14 057 (85.6)	7994 (57.9)
	Depression	13 436 (2.5)	10 986 (19.8)	786 (4.8)	2668 (19.3)
	Other	36 164 (6.6)	13 040 (23.5)	1577 (9.6)	3142 (22.8)
**Pregnancy outcomes**					
Pre-term delivery (<37 weeks)	No	520 445 (95.4)	51 649 (93.0)	15 640 (95.2)	12 868 (93.2)
	Yes	25 122 (4.6)	3890 (7.0)	780 (4.8)	936 (6.8)
Offspring malformation	No	535 160 (98.1)	54 244 (97.7)	16 104 (98.1)	13 492 (97.7)
	Yes	10 407 (1.9)	1295 (2.3)	316 (1.9)	312 (2.3)
Stillbirth or neonatal death	No	543 416 (99.6)	54 987 (99.0)	16 363 (99.7)	13 681 (99.1)
	Yes	2151 (0.4)	552 (1.0)	57 (0.3)	123 (0.9)

BMI, body mass index; cig., cigarettes; *N*, number; PND, perinatal depression; SD, standard deviation.

^a^Missing values (*n* = 257) included in Q1.

### Statistical analysis

Data were cleaned using Stata (version 17 IQT) and analysed using SAS (version 9.4).

#### Main analysis

We used stratified Cox model, conditioned on matching set, to examine the association between PND and subsequent CVD using hazard ratios (HRs) with 95% confidence intervals (CIs). We found no evidence of violation of assumption of proportional hazards over time when conducting a Schoenfeld residuals test.^25^ In all models, we applied the clustered sandwich estimator, which allows for the standard errors for intragroup correlation, relaxing the requirement that the observations be independent. We also performed analyses for APD and PPD separately. To assess potential heterogeneity of the associations according to CVD subtypes, we conducted analyses for type-specific CVDs.

We developed three models. In the first model, we adjusted for age and calendar year of delivery (by stratifying on matching set). In the second model, we additionally adjusted for country of birth, marital status, education, and income. In the last model, we additionally adjusted for smoking before pregnancy, BMI in early pregnancy, parity, diabetes, pre-eclampsia, and history of psychiatric disorders.

To address unmeasured confounders such as genetic and familial environmental factors (e.g. childhood abuse, parental mental ill-health, and stress in the family), we also conducted a sibling-matched cohort. Briefly, we restricted all PND women in the study base to those who had at least one full and parous sister in our data (*n* = 14 446), identified from the Swedish Multi-Generation Register, and randomly sampled one PND-free pregnancy from each sister of these PND women for comparison (*n* = 29 423). We then excluded women with PND who had a CVD before the index date (*n* = 445) and their unaffected sisters (*n* = 886), and further, we excluded unaffected sisters who had a CVD before the index date (*n* = 540). Hence, we included 13 804 women with PND (5925 APD and 7879 PPD) and their 16 420 unaffected sisters. The follow-up started from the date of PND diagnosis for the PND women and the same gestational day/postpartum day for the unaffected sisters. The Cox model was then conditioned on sibling set to contrast the risk of CVD between PND women and their PND-free sisters.

#### Additional analyses

We employed Poisson regression to estimate relative risk (RR) of CVD within 5, 10, 15, and 20 years after matching. To assess the impact of comorbidities, where some can also be considered mediators of the association between APD and CVD, we conducted stratified analyses by history of psychiatric disorders, pre-eclampsia, and diabetes. To investigate the role of subsequent episodes of major depression, we performed stratified analyses on the diagnosis of depression or filled prescription of antidepressants, if any, beyond 1 year post-partum. The subsequent depressive episode was treated as timing-varying. For example, a PND or unaffected woman contributed person-times to no subsequent episode group until receiving a subsequent depression diagnosis or prescription and then contributed to the subsequent episode group. These analyses were also repeated in the sibling comparison.

To investigate potential effect modification by well-known CVD risk factors, we stratified the analyses by age, smoking, and BMI categories. Additionally, we stratified the analysis by calendar period as the missed diagnoses of PND could be lower in recent years with increased awareness and the introduction of screening programmes and by parity as parity has been suggested to be related to CVD^26^ and PND.^27^ An interaction term between PND and each stratification variable was added and tested for statistical significance as *P* for interaction.

To provide insights into the importance of the timing of PND diagnosis, we conducted analyses for PND diagnosed during 0–13 weeks and ≥14 weeks of gestation and during 0–3 months, 4–6 months, or 7–12 months postpartum. Additionally, to shed light on the temporal pattern of the association, we examined the associations by different periods of follow-up, including <1, 1–4, 5–9, and ≥ 10 years from index date.

To test the robustness of our findings, we performed sensitivity analyses where we limited the analyses to (i) PND identified through diagnoses alone, (ii) CVD identified through primary diagnosis alone, (iii) CVD not including hypertensive disease, or (iv) women without adverse pregnancy outcomes. Further, in a sensitivity analysis, we included any prescription of antihypertensives (ATC codes C02-C03 and C07-C09 except C09BX) to complement the identification of hypertension in the patient register. Due to data availability, this analysis was restricted to women born in Sweden from 1973 onwards, giving birth during 2006–14. In this analysis, women with this complemented definition of hypertension before index date were excluded.

## Results

The mean age at diagnosis of PND was 30.8 (standard deviation 5.6) years. Compared with women without PND (*n* = 545 567), women with PND (*n* = 55 539) were more likely to be born in Sweden, to be married, and to have a lower educational attainment. They were more likely to smoke before pregnancy, to have a history of depression, and to deliver pre-term or by caesarean section (*Table 1*).

During a median follow-up of 10.4 years (maximum 20), we observed 3533 (6.1 per 1000 person-years) and 20 202 (3.6 per 1000 person-years) first diagnoses of CVD among women with and without PND, respectively. Compared with women without PND, women with PND had a 36% higher risk of any CVD (fully adjusted HR 1.36, 95% CI 1.31–1.42; *Table 2*). The higher risk was lower for APD (HR 1.29, 95% CI 1.22–1.37) than for PPD (HR 1.42, 95% CI 1.35–1.49). In the sibling comparison, the association was attenuated by 44% but remained significant in the fully adjusted model (HR 1.20, 95% CI 1.07–1.34; *Table 2*). The relative risk was comparable in varying lengths of follow-up (RR 1.42–1.47; Supplementary data online, *Table S2*) and with the HRs in *Table 2*.

**Table 2:** Association of perinatal depression with risk of cardiovascular disease: a nationwide population-matched cohort and a sibling-matched cohort, 2001–20

	CVD			Model 1	Model 2	Model 3
	*N*, obs.	*N*, exp.	IR	HR (95% CI)^a^	HR (95% CI)^b^	HR (95% CI)^c^
**Population-matched cohort**						
**No PND**	20 202	20 202	3.6	1.00	1.00	1.00
**PND**	3533	2077	6.1	1.70 (1.64–1.76)	1.62 (1.56–1.67)	1.36 (1.31–1.42)
Antepartum	1575	905	6.2	1.70 (1.62–1.79)	1.61 (1.53–1.69)	1.29 (1.22–1.37)
Post-partum	1958	1702	6.0	1.70 (1.63–1.78)	1.62 (1.55–1.70)	1.42 (1.35–1.49)
**Sibling-matched cohort**						
**No PND**	878	878	4.7	1.00	1.00	1.00
**PND**	856	682	5.9	1.28 (1.16–1.41)	1.24 (1.12–1.36)	1.20 (1.07–1.34)
Antepartum	383	293	6.0	1.39 (1.20–1.59)	1.35 (1.17–1.55)	1.27 (1.08–1.49)
Post-partum	473	389	5.8	1.21 (1.08–1.36)	1.17 (1.04–1.32)	1.16 (1.02–1.32)

CI, confidence interval; CVD, cardiovascular disease; HR, hazard ratio; IR, incidence rate per 1000 person-years; *N*, number; obs, number of CVD events; exp, expected number of CVD events if the women were not exposed to PND and this estimate was based on the exposure group in the pooled sample instead of within matching set; PND, perinatal depression.

^a^Hazard ratios were adjusted for age and calendar year (through stratifying on risk set in the population-matched comparison). In the sibling-matched cohort, the analysis was stratified on sibling set.

^b^Hazard ratios were additionally adjusted for country of birth, marital status, education, and income.

^c^Hazard ratios were additionally adjusted for smoking before pregnancy, body mass index in early pregnancy, parity, diabetes, pre-eclampsia, and history of psychiatric disorder.

The association between PND and CVD was strongest in women without psychiatric comorbidities (*P* for interaction < .001; *Table 3*). To test the specificity of the studied association, we estimated HRs using women without PND and psychiatric comorbidities as a constant reference (see Supplementary data online, *Table S3*) and found a similar effect size for the association between the PND and CVD risk (HR 1.43) to that observed in the case of major depression and CVD risk (HR 1.62). Notably, PND was associated with a higher risk of CVD among women with a prior depression (HR 1.72) or other psychiatric disorders (HR 1.82), compared with women without such history. When stratifying on depression developed beyond 1 year postpartum, an increased risk of CVD was noted in women with no subsequent depression (HR 1.32, 95% CI 1.22–1.42; see Supplementary data online, *Table S4*). Comparable results were otherwise found between women with and without pre-eclampsia (*P* for interaction > .05; *Table 3*), whereas women with gestational diabetes displayed higher risk (HR 1.80, 95% CI 1.44–2.26) compared with women without diabetes (HR 1.36, 95% CI 1.30–1.41, *P* for interaction = .028; *Table 3*). Comparable results were found across groups by age, smoking, BMI, calendar year, and parity categories (*P* for interaction > .05; Supplementary data online, *Table S5*), whereas no clear trend was shown for smoking (*P* for interaction = .02; see Supplementary data online, *Table S5*).

**Table 3:** Associations of perinatal depression with risk of cardiovascular disease, stratified by psychiatric and pregnancy comorbidities: a nationwide population-matched cohort, 2001–20

	No PND	PND		Antepartum depression		Post-partum depression	
	CVD	CVD	HR (95% CI)^a^	CVD	HR (95% CI)^a^	CVD	HR (95% CI)^a^
	*N* (IR)	*N* (IR)		*N* (IR)		*N* (IR)	
**History of psychiatric disorder**							
No	18 070 (3.5)	1840 (5.5)	1.43 (1.37–1.50)	646 (5.4)	1.37 (1.26–1.48)	1194 (5.6)	1.47 (1.39–1.56)
Depression	600 (5.5)	775 (6.9)	1.06 (0.94–1.20)	528 (7.0)	1.04 (0.91–1.19)	247 (6.7)	1.10 (0.94–1.29)
Other	1532 (4.6)	918 (6.9)	1.36 (1.24–1.49)	401 (7.0)	1.32 (1.17–1.48)	517 (6.8)	1.39 (1.25–1.55)
*P* for interaction^b^	<.001			.003		.004	
**Pre-eclampsia**							
No	18 550 (3.4)	3158 (5.7)	1.36 (1.31–1.42)	1382 (5.7)	1.28 (1.20–1.36)	1776 (5.6)	1.42 (1.35–1.50)
Yes	1652 (9.8)	375 (16.4)	1.37 (1.21–1.55)	193 (17.8)	1.38 (1.17–1.64)	182 (15.1)	1.34 (1.14–1.59)
*P* for interaction^b^	.931			.392		.530	
**Diabetes**							
No	19 282 (3.5)	3280 (5.8)	1.36 (1.30–1.41)	1447 (5.9)	1.28 (1.21–1.36)	1833 (5.7)	1.41 (1.34–1.48)
Gestational	434 (7.2)	120 (14.6)	1.80 (1.44–2.26)	59 (14.4)	1.72 (1.26–2.33)	61 (14.8)	1.88 (1.41–2.51)
Pre-gestational	486 (15.2)	133 (23.9)	1.20 (0.95–1.51)	69 (26.7)	1.20 (0.88–1.63)	64 (21.4)	1.20 (0.89–1.62)
*P* for interaction^b^	.028			.162		.084	

CI, confidence interval; CVD, cardiovascular disease; HR, hazard ratio; IR, incidence rate, per 1000 person-years; *N*, number; PND, perinatal depression.

^a^Hazard ratios were adjusted for age and calendar year (through stratifying on risk set), country of birth, marital status, education, income, smoking before pregnancy, body mass index in early pregnancy, parity, diabetes, pre-eclampsia, and history of psychiatric disorder, whenever applicable.

^b^The *P*-value corresponds to the interaction term between PND and the specific stratification factor.

When studying the associations according to CVD subtypes, we found that PND was positively associated with all subtypes, with the greatest HR for hypertensive disease (HR 1.50, 95% CI 1.41–1.60; *Table 4*), ischaemic heart disease (HR 1.37, 95% CI 1.13–1.65; *Table 4*), and heart failure (HR 1.36, 95% CI 1.06–1.74; *Table 4*). With overlapping CIs, the association with ischaemic heart disease and heart failure appeared more prominent for APD than for PPD, whereas the associations with other CVD subtypes were more prominent for PPD (*Table 4*).

**Table 4:** Associations of perinatal depression with cardiovascular disease subtypes: a nationwide population-matched cohort, 2001–20

	No PND	PND		Antepartum depression		Post-partum depression	
	CVD	CVD	HR (95% CI)^a^	CVD	HR (95% CI)^a^	CVD	HR (95% CI)^a^
	*N* (IR)	*N* (IR)		*N* (IR)		*N* (IR)	
**Hypertensive disease**	7771 (1.4)	1581 (2.7)	1.50 (1.41–1.60)	719 (2.8)	1.44 (1.31–1.58)	862 (2.6)	1.55 (1.43–1.67)
Essential hypertension	7556 (1.3)	1547 (2.6)	1.51 (1.42–1.61)	699 (2.7)	1.45 (1.32–1.60)	848 (2.5)	1.56 (1.44–1.69)
Other	461 (0.1)	74 (0.1)	1.21 (0.90–1.63)	43 (0.2)	1.20 (0.83–1.73)	31 (0.1)	1.22 (0.81–1.85)
**Ischaemic heart disease**	833 (0.1)	172 (0.3)	1.37 (1.13–1.65)	83 (0.3)	1.44 (1.10–1.89)	89 (0.3)	1.31 (1.03–1.66)
**Cerebrovascular disease**	1354 (0.2)	215 (0.4)	1.05 (0.89–1.23)	102 (0.4)	1.04 (0.82–1.31)	113 (0.3)	1.05 (0.86–1.28)
Subarachnoid haemorrhage	310 (0.1)	41 (0.1)	0.82 (0.56–1.20)	22 (0.1)	0.99 (0.59–1.66)	19 (0.1)	0.70 (0.43–1.14)
Haemorrhagic stroke	299 (0.1)	57 (0.1)	1.23 (0.90–1.69)	21 (0.1)	0.95 (0.57–1.59)	36 (0.1)	1.44 (1.00–2.08)
Ischaemic stroke	832 (0.1)	125 (0.2)	1.03 (0.83–1.27)	61 (0.2)	1.04 (0.78–1.39)	64 (0.2)	1.02 (0.78–1.32)
Other	1108 (0.2)	185 (0.3)	1.16 (0.97–1.39)	88 (0.3)	1.13 (0.88–1.46)	97 (0.3)	1.18 (0.95–1.47)
**Emboli and thrombosis**	1739 (0.3)	331 (0.6)	1.33 (1.16–1.52)	147 (0.6)	1.18 (0.97–1.44)	184 (0.5)	1.45 (1.24–1.70)
**Heart failure**	554 (0.1)	103 (0.2)	1.36 (1.06–1.74)	56 (0.2)	1.38 (1.00–1.92)	47 (0.1)	1.33 (0.97–1.83)
**Arrhythmias**	7274 (1.3)	1120 (1.9)	1.28 (1.20–1.37)	486 (1.9)	1.22 (1.10–1.34)	634 (1.9)	1.33 (1.22–1.45)
Cardiac arrest	6533 (1.1)	976 (1.6)	1.26 (1.17–1.36)	427 (1.7)	1.22 (1.09–1.35)	549 (1.6)	1.29 (1.18–1.41)
Arrhythmia	809 (0.1)	104 (0.2)	1.03 (0.82–1.29)	49 (0.2)	0.99 (0.72–1.37)	55 (0.2)	1.06 (0.80–1.41)
Conduction disorder	246 (0.0)	89 (0.1)	2.31 (1.75–3.04)	35 (0.1)	1.65 (1.09–2.50)	54 (0.2)	2.85 (2.08–3.92)
**Other CVDs**	2921 (0.5)	494 (0.8)	1.37 (1.23–1.52)	212 (0.8)	1.20 (1.03–1.40)	282 (0.8)	1.50 (1.33–1.71)

CI, confidence interval; CVD, cardiovascular disease; HR, hazard ratio; IR, incidence rate, per 1000 person-years; *N*, number; PND, perinatal depression.

^a^Hazard ratios were adjusted for age and calendar year (through stratifying on risk set), country of birth, marital status, education, income, smoking before pregnancy, body mass index in early pregnancy, parity, diabetes, pre-eclampsia, and history of psychiatric disorder.

When studying the importance of the timing of PND diagnosis, we observed positive associations for all timepoints, with the strongest association shown for PND diagnosed 4–6 months postpartum (HR 1.46, 95% CI: 1.33–1.61; see Supplementary data online, *Table S6*). Regarding time since diagnosis, similar results were found over time, with the greatest HR noted 1–4 years after PND diagnosis (see Supplementary data online, *Table S7*).

Similar associations were yielded in the sensitivity analyses limiting to clinical diagnoses of PND, primary diagnoses of CVD, CVD not including hypertensive disease, or women without adverse pregnancy outcomes (see Supplementary data online, *Table S8*). We found comparable associations when complementing hypertension with prescriptions of antihypertensives (HR 1.90, 95% CI 1.84–1.96; Supplementary data online, *Table S9*).

## Discussion

In this Swedish nationwide register-based study with a follow-up of up to 20 years, we found that women with clinically diagnosed PND were at a higher risk of CVD compared with matched unaffected women (*Structured Graphical Abstract*). The association was stronger for PPD than for APD. We found largely comparable results, particularly for PPD, when comparing women with and without a history of psychiatric disorders, although not significantly for depression, indicating that the association cannot be explained by psychiatric comorbidities alone. The attenuated association noted in the sibling comparison suggests a sizable contribution of familial factors (e.g. genetics or early environmental factors) to the observed association although that they cannot completely explain the association. Our findings may help healthcare providers to identify high-risk women and predict CVD development for early detection and intervention.

To the best of our knowledge, the present study is the first full report to show an association between PND and subsequent CVD risk. In a conference abstract, Divanji *et al*.^28^ reported a 67% (HR 1.67, 95% CI 1.37–2.03) risk increase of overall CVD in women with PPD in the USA during a mean follow-up of 5.2 years. Although the results are in line with our finding on PPD (HR 1.42), the limited information provided on the design and methods (e.g. adjustment of covariates) of this previous study did not allow us to compare our studies. We are not aware of any report on the risk of CVD among women with APD. Future research is needed to confirm our findings.

Although CIs were overlapping among the different subtypes, we found that the most pronounced association was seen for ischaemic heart disease, hypertensive disease, and heart failure. In line with our study, a 23% increased risk of heart failure and a 56% increased risk of ischaemic heart disease has been estimated for patients with non-perinatal major depression in meta-analyses.^29,30^ In Sweden, hypertension is often managed in primary care and is therefore not completely captured by the NPR. That said, hypertensive disease identified in our study is likely the severe cases, not responding well to first-line treatment, or having other comorbidities to be attended by specialized care. However, we found comparable associations when complementing using prescriptions of antihypertensives. Future studies including hypertensive disease attended in primary care are needed.

The risk of developing CVD seems slightly different between women with APD and PPD. The association with overall and type-specific CVD, except for ischaemic heart disease and heart failure, was constantly higher for PPD than for APD. These findings suggest separate trajectories of CVD development depending on the timing of PND onset, although the diagnosis of PND is likely delayed in registers. Differences in biological mechanisms and aetiology^18,19^ may contribute to the observed difference in CVD risk for APD and PPD, where hormonal^31^ and immune system alterations during pregnancy and postpartum^32^ might play a role.^33,34^

There are several explanations for our findings. Emerging evidence has revealed shared genetic makeup (e.g. the 20q12 as a pleiotropic region and the *RPL31P12*, *BORSC7*, *PNPT11*, and *PGF* as specific genes) between major depression and CVD.^10,35^ It is therefore plausible that genetic factors also underlie the link between PND and CVD. Indeed, the association was attenuated in the sibling comparison where genetics are partially controlled for. Shared familial environmental factors, such as childhood maltreatment,^36^ may also partly explain this attenuation in sibling comparison. We encourage further research to better understand the contributing factors or mechanisms.

Depression, with a 2:1 female-to-male ratio,^37^ and other psychiatric disorders outside the perinatal period have been linked to both PND^27,38,39^ and CVD.^10,40–43^ However, we found that the association noted in the present study was not explained by psychiatric comorbidities. Previous studies often exclude women with a history of psychiatric disorders, resulting in limited evidence available for this group. By assessing both women with and without a history of psychiatric disorders, we not only demonstrate that women who experienced their first depressive episode as a PND are at increased risk of CVD but also that PND adds to the risk of CVD in women with a previous psychiatric disorder other than depression. Of note, among women with a history of depression, PND was not significantly associated with an increased risk of CVD. We further showed that the association between PND and the risk of CVD was largely comparable with that between non-PND and the risk of CVD, indicating the importance of screening for history of both PND and non-PND in the assessment of CVD risk. Non-PND has been linked to factors involved in the pathogenesis of CVD^44^ including inflammation,^45^ oxidative stress,^46,47^ stress-related neuroendocrine and/or cardiometabolic changes,^48,49^ behavioural/lifestyle changes,^50^ and recurrent psychiatric episodes.^51^ Likewise, PND may lead to future psychiatric conditions^52^ and use of psychotropic medications which could mediate the association we found for PND and CVD.^53^ In fact, some antidepressants demonstrate cardioprotective effect while others have, when used chronically, the opposite effect.^54^ However, the positive association remained between PND and CVD among women with no subsequently recorded depressive episodes, suggesting that our findings are not completely explained by the subsequent development of major depression. We encourage future studies investigating the mediating roles of subsequent depressive episode and antidepressant treatment. In addition, PND has also been linked to oxidative stress^55–59^ and other oxidative-inflammatory traits, such as smoking,^60^ high BMI and high weight retention after delivery,^61,62^ adverse health behaviours,^63^ and secondary unhealthy lifestyle.^64^ These factors have previously been discussed for PND in relation to CVD in pregnancy, such as pre-eclampsia and peripartum cardiomyopathy.^11^ It is possible that stress-related neuroendocrine and/or cardiometabolic changes play a role in the link between PND and CVD. Future research is warranted to understand the disease pathways leading to CVD, including both women with and without psychiatric comorbidities.

Perinatal depression has also been linked to pregnancy complications, such as pre-eclampsia^8^ and gestational diabetes,^7^ which in turn are associated with a higher risk of CVD after the delivery.^65,66^ While the incidence rate of CVD was higher for women with pre-eclampsia or gestational diabetes than among those free of these conditions, our findings based on the stratified analysis suggest that the association between PND and CVD is independent of these pregnancy complications. Our findings may have strong clinical and public health relevance by suggesting the importance of factoring in PND for risk prediction or stratification of future CVD events among women.

### Strengths and limitations

The major strengths of this study include the nationwide population-based design with prospectively collected data on a rich set of phenotypes including covariables and the complete and long (up to 20 years) follow-up. The large sample size allows us to perform stratified analyses such as for timing of diagnoses and for specific outcomes and gives us the possibility to conduct sibling analyses. This study also has limitations. First, using the PND definition of the present study, we could not ascertain PND diagnosed after 2014, PND diagnosed in primary care without prescription of antidepressants, and women with PND symptoms who did not seek for healthcare service. However, such misclassification (classifying PND women as unaffected women) would have led to attenuated associations. There is also a risk that the onset of PND is before conception, although it was diagnosed or treated during or after pregnancy. However, the analyses concerning the timing of PND diagnosis yielded similar results across timepoints, assuming that APD diagnosed/treated at or after 14 gestational weeks is less likely to have an onset before conception. Second, antidepressants can be used for reasons other than depression. However, comparable findings were obtained when only including clinical diagnoses of PND as ascertainment of PND. Third, there is also a risk of misclassification of CVD as we included secondary diagnoses in the definition of CVD, which are less validated. Still, when restricting to primary diagnoses of CVD, the results remained similar to those observed in the main analyses. Further, women with CVD treated in primary care (e.g. those with hypertension) might have been missed in the ascertainment of CVD outcomes. However, the most severe CVDs, in addition to the regular treatment in primary care, are diagnosed and treated within specialist care and, hence, captured by the registers, and comparable results were shown when we excluded hypertensive disorders from CVD. In addition, women diagnosed with PND might have more contacts with healthcare providers. As a result, CVD might be more frequently detected for these women. Yet, a similar risk increase was noted 5–9 years after the PND diagnosis, arguing against the possibility of surveillance bias being the pure explanation. Further, bias by residual confounding, including for example diet and socioeconomic status other than income and education, is possible, although we extensively adjusted for confounders in multivariable models and through the sibling design. Last, the mean age of our study sample was 41 years at the end of follow-up, which is before the peak age of CVD in Sweden. Future studies with longer follow-up into late adulthood are needed.

## Conclusions

Women with clinically diagnosed PND had a higher rate of CVD in mid-adulthood. Although familial factors may partly play a role here, our findings lend support to the ongoing discussion on factoring in reproductive history, including PND, for CVD risk assessment and prediction in women.

## Supplementary Material

ehae170_Supplementary_Data

## Data Availability

Due to Swedish privacy protection governed by the General Data Protection Regulation, access to register data can only be granted after ethical approval by the ethics review authority. Information can be found at the Swedish National Board of Health and Welfare (https://bestalladata.socialstyrelsen.se/, email: registerservice@socialstyrelsen.se) and/or Statistics Sweden (https://www.scb.se/vara-tjanster/bestall-data-och-statistik/, email: scb@scb.se). Upon reasonable request can codes for data analysis be shared.

## Pre-registered Clinical Trial Number

None supplied.
