# Supplementary material for: Perinatal depression and risk of maternal cardiovascular disease: a Swedish nationwide study
Source: Eur Heart J. 2024 Jun 18;45(31):2865–75. doi: 10.1093/eurheartj/ehae170 (PMC11328867; doi:10.1093/eurheartj/ehae170)
Supplement: ehae170_Supplementary_Data [file ehae170_supplementary_data.docx]

**Supplementary Table 1.** International Statistical Classification of Diseases -codes

|  | **ICD-9** | | **ICD-10** |
| --- | --- | --- | --- |
| ***Psychiatric diseases*** | | | |
| Perinatal depression | 311 | | F530, F32-F33 |
| History of depression | 300E, 311 | | F32-F39 |
| History of other psychiatric disorders | 295-319 (excluding: 300E, 311) | | F10-F99 (excluding: F32-F39) |
| ***Pregnancy diseases*** | | | |
| Gestational diabetes | 648W | | O224 |
| Pregestational diabetes | 250 | | O240- O243, E10-E14 |
| Preeclampsia | - | | O14, O15 |
| ***Child characteristics*** |  | |  |
| Major malformations | 740-759, excluding:  744B, 744C, 744E, 747A, 747F, 748D, 750A, 752E, 752F, 754D, 754G, 755A, 755B, 756B, 756X, 757C | | Q00-Q99 (excluding:  Q170, Q175, Q180, Q181, Q250, Q270, Q314, Q320, Q381, Q523, Q530-Q539, Q650-Q659, Q665-Q669, Q690, Q699, Q703, Q760, Q799, Q825, Q829) |
| ***Cardiovascular diseases*** | | | |
| **Any CVD** | 390-438, 440,444,445 | I00-I70, I730, I74- I75 | |
| **Hypertensive diseases** |  |  | |
| Essential hypertension | 401 | I10 | |
| Other hypertensive disease | 402-405 | I11- I16, I674 | |
| **Ischemic heart disease** | 410-414 | I20-I25 | |
| **Cerebrovascular disease** |  |  | |
| Subarachnoid hemorrhage | 430 | I60 | |
| Hemorrhagic stroke | 431-432 | I61-I62 | |
| Ischemic stroke | 433, 434 | I63 | |
| Other cerebrovascular disease | 436-438 | I65-I69, I674 | |
| **Emboli and thrombosis** | 415, 444-445 | I26, I74-I75 | |
| **Heart failure** | 428 | I50 | |
| **Arrhythmia/conduction disorder** |  |  | |
| Arrhythmia | 427 (excl.427F) | I47-I49 | |
| Conduction disorder | 426 | I44-I45 | |
| Cardiac arrest | 427F | I46 | |

ICD = International Statistical Classification of Diseases, CVD = cardiovascular disease

**Supplementary Table 2.** Relative risk of cardiovascular disease among women with perinatal depression within 5, 10, 15, or 20 years after the matching.

|  | | **No PND** | **PND** | | **Antepartum depression** | | **Postpartum depression** | | |
| --- | --- | --- | --- | --- | --- | --- | --- | --- | --- |
|  | | **(n= 545,567)** | **(n=55,539)** | | **(n= 23,871)** | | **(n= 31,668)** | | |
| Follow-up (yr) | | CVD, N (IR) | CVD, N (IR) | RR (95% CI) | CVD, N (IR) | RR (95% CI) | CVD, N (IR) | RR (95% CI) | |
| 5 | | 7,562 (1.4) | 1,366 (2.5) | 1.47 (1.38-1.57) | 605 (2.5) | 1.33 (1.21-1.47) | 761 (2.4) | 1.58 (1.46-1.72) | |
| 10 | | 15,348 (2.8) | 2,710 (4.9) | 1.42 (1.35-1.48) | 1,214 (5.1) | 1.35 (1.26-1.45) | 1,496 (4.7) | 1.47 (1.38-1.55) | |
| 15 | | 19,687 (3.6) | 3,450 (6.2) | 1.42 (1.36-1.48) | 1,538 (6.4) | 1.39 (1.31-1.48) | 1,912 (6.0) | 1.44 (1.37-1.51) | |
| 20 | | 20,202 (3.7) | 3,533 (6.4) | 1.42 (1.36-1.48) | 1,575 (6.6) | 1.39 (1.31-1.47) | 1,958 (6.2) | 1.44 (1.37-1.51) | |
| CI, confidence interval; CVD, cardiovascular disease; RR, relative risk; IR, incidence rate, per 1000 person-years; N, number; PND, perinatal depression. | | | | | | | |  |  |
| RRs were adjusted for age and calendar year (through stratifying on risk set), country of birth, marital status, education, income, smoking before pregnancy, body mass index in early pregnancy, parity, diabetes, preeclampsia, and history of psychiatric disorder. | | | | | | | |  |  |

| **Supplementary Table 3.** Association between perinatal depression, history of psychiatric disorder and risk cardiovascular disease. | | | |
| --- | --- | --- | --- |
|  |  |  |  |
| **History of psychiatric disorder** | **PND** | **CVD** | **HR (95% CI)** |
|  |  | **N (IR)** |  |
| No | No PND | 18,070 (3.5) | 1.00 |
| No | PND | 1,840 (5.5) | 1.43 (1.37-1.50) |
|  | Antepartum | 646 (5.4) | 1.37 (1.26-1.48) |
|  | Postpartum | 1,194 (5.6) | 1.47 (1.39-1.56) |
| Depression | No PND | 600 (5.5) | 1.62 (1.46-1.80) |
|  | PND | 775 (6.9) | 1.72 (1.60-1.85) |
|  | Antepartum | 528 (7.0) | 1.69 (1.55-1.85) |
|  | Postpartum | 247 (6.7) | 1.78 (1.57-2.03) |
| Other disorder | No PND | 1,532 (4.6) | 1.34 (1.26-1.44) |
|  | PND | 918 (6.9) | 1.82 (1.70-1.95) |
|  | Antepartum | 401 (7.0) | 1.77 (1.60-1.96) |
|  | Postpartum | 517 (6.8) | 1.87 (1.71-2.04) |
| CI, confidence interval; CVD, cardiovascular disease; HR, hazard ratio; IR, incidence rate, per 1000 person-years; N, number; PND, perinatal depression. | | | |
| HRs were adjusted for age and calendar year (through stratifying on risk set), country of birth, marital status, education, income, smoking before pregnancy, body mass index in early pregnancy, parity, diabetes, preeclampsia, and history of psychiatric disorder, whenever applicable. | | | |

**Supplementary Table 4.** Associations of perinatal depression with risk cardiovascular disease stratified by subsequent depressive episode, defined as a diagnosis of depression or filled prescription of antidepressants beyond one year postpartum.

| **Population** | **No PND** | **PND** | |  | **Antepartum depression** | |  | **Postpartum depression** | |  |
| --- | --- | --- | --- | --- | --- | --- | --- | --- | --- | --- |
| Subsequent depressive episode | CVD, N(IR) | CVD, N(IR) | HR (95% CI) | p-for interaction | CVD, N(IR) | HR (95% CI) | p-for interaction | CVD, N(IR) | HR (95% CI) | p-for interaction |
| No | 15,436 (3.2) | 815 (4.8) | 1.32 (1.22-1.42) | <0.001 | 445 (5.0) | 1.20 (1.08-1.32) | 0.003 | 370 (4.4) | 1.49 (1.34-1.65) | <0.001 |
| Yes | 4,766 (6.1) | 2,718 (6.7) | 1.02 (0.97-1.07) | | 1,130 (6.9) | 1.00 (0.93-1.08) |  | 1,588 (6.5) | 1.03 (0.97-1.09) | |
| **Sibling** | **No PND** | **PND** | |  | **Antepartum depression** | |  | **Postpartum depression** | |  |
| Subsequent depressive episode | CVD, N(IR) | CVD, N(IR) | HR (95% CI) | p-for interaction | CVD, N(IR) | HR (95% CI) | p-for interaction | CVD, N(IR) | HR (95% CI) | p-for interaction |
| No | 547 (3.9) | 412 (5.4) | 1.31 (1.13-1.51) | 0.063 | 183 (5.6) | 1.29 (1.05-1.59) | 0.583 | 229 (5.2) | 1.33 (1.11-1.59) | 0.028 |
| Yes | 331 (6.8) | 444 (6.4) | 1.04 (0.87-1.24) | | 200 (6.5) | 1.19 (0.93-1.51) |  | 244 (6.4) | 0.96 (0.79-1.18) | |
| CI, confidence interval; CVD, cardiovascular disease; HR, hazard ratio; IR, incidence rate, per 1000 person-years; N, number; PND, perinatal depression. | | | | | | | | | | |
| HRs were adjusted for age and calendar year (through stratifying on risk set), country of birth, marital status, education, income, smoking before pregnancy, body mass index in early pregnancy, parity, diabetes, preeclampsia, and history of psychiatric disorder, whenever applicable. | | | | | | | | | | |

| **Supplementary Table 5. Associations of perinatal depression with risk cardiovascular disease, stratified by age, calendar year, smoking, and BMI.** | | | | | | | | | |
| --- | --- | --- | --- | --- | --- | --- | --- | --- | --- |
|  | **No PND** | **PND** | | **Antepartum depression** | | | **Postpartum depression** | | |
|  | CVD | CVD | HR (95% CI) ^a^ | CVD | HR (95% CI) ^a^ | | CVD | | HR (95% CI) ^a^ |
|  | N (IR) | N (IR) |  | N (IR) |  |  | N (IR) | |  |
| **Age at matching, years** | | | |  |  | |  | |  |
| <20 | 236 (2.7) | 44 (4.7) | 1.36 (0.98-1.88) | 23 (4.2) | 1.09 (0.72-1.66) | | 21 (5.4) | | 1.83 (1.12-3.00) |
| 20-24 | 1,933 (2.7) | 361 (4.8) | 1.47 (1.31-1.65) | 182 (5.1) | 1.48 (1.26-1.74) | | 179 (4.6) | | 1.44 (1.23-1.69) |
| 25-29 | 4,342 (2.9) | 775 (5.0) | 1.40 (1.30-1.52) | 362 (5.2) | 1.39 (1.24-1.55) | | 413 (4.8) | | 1.41 (1.27-1.56) |
| 30-34 | 6,099 (3.3) | 1,079 (5.7) | 1.39 (1.30-1.48) | 475 (6.0) | 1.30 (1.18-1.43) | | 604 (5.5) | | 1.45 (1.34-1.58) |
| ≥35 | 7,592 (5.2) | 1,274 (8.4) | 1.29 (1.22-1.37) | 533 (8.6) | 1.18 (1.08-1.30) | | 741 (8.2) | | 1.37 (1.27-1.48) |
| P for interaction ^b^ | 0.224 | | | 0.064 | | 0.717 | | | |
| **Calendar year at matching** | | | |  |  | |  | |  |
| 2001-2004 | 1,974 (4.5) | 301 (7.7) | 1.34 (1.19-1.51) | 164 (8.3) | 1.42 (1.20-1.66) | | 137 (7.1) | | 1.25 (1.06-1.49) |
| 2005-2009 | 11,163 (3.8) | 2,002 (6.6) | 1.37 (1.30-1.44) | 842 (6.6) | 1.28 (1.19-1.38) | | 1,160 (6.6) | | 1.44 (1.35-1.53) |
| 2010-1014 | 7,065 (3.1) | 1,230 (5.2) | 1.35 (1.27-1.44) | 569 (5.5) | 1.27 (1.16-1.39) | | 661 (5.0) | | 1.42 (1.31-1.54) |
| P for interaction ^b^ | 0.918 | | | 0.486 | | 0.339 | | | |
| **Smoking before pregnancy** | | | |  |  | |  | |  |
| No | 15,723 (3.5) | 2,183 (5.6) | 1.31 (1.25-1.38) | 967 (5.8) | 1.26 (1.17-1.35) | | 1,216 (5.4) | | 1.35 (1.27-1.43) |
| 1-9 cig./day | 1,544 (3.5) | 410 (6.3) | 1.52 (1.35-1.70) | 180 (6.2) | 1.37 (1.17-1.61) | | 230 (6.4) | | 1.64 (1.42-1.89) |
| ≥10 cig./day | 1,925 (4.4) | 754 (7.7) | 1.41 (1.28-1.55) | 345 (7.5) | 1.28 (1.13-1.46) | | 409 (8.0) | | 1.52 (1.36-1.71) |
| Unknown | 1,010 (3.8) | 186 (6.9) | 1.58 (1.35-1.86) | 83 (7.6) | 1.65 (1.31-2.08) | | 103 (6.4) | | 1.52 (1.24-1.87) |
| P for interaction ^b^ | 0.020 | | | 0.131 | | 0.037 | | | |
| **BMI, kg/m^2^** |  |  |  |  |  | |  | |  |
| <18.5 | 307 (2.5) | 64 (4.7) | 1.56 (1.18-2.06) | 26 (4.3) | 1.36 (0.91-2.05) | | 38 (5.1) | | 1.71 (1.21-2.42) |
| 18.5-24.9 | 9,141 (2.9) | 1,327 (4.6) | 1.34 (1.26-1.43) | 600 (4.9) | 1.34 (1.23-1.47) | | 727 (4.3) | | 1.34 (1.24-1.44) |
| 25-29.9 | 4,930 (3.8) | 903 (6.5) | 1.43 (1.33-1.54) | 375 (6.0) | 1.22 (1.09-1.36) | | 528 (6.9) | | 1.62 (1.47-1.78) |
| ≥30 | 3,877 (6.5) | 862 (10.3) | 1.26 (1.16-1.37) | 397 (10.3) | 1.17 (1.04-1.31) | | 465 (10.4) | | 1.34 (1.21-1.49) |
| Unknown | 1,947 (4.0) | 377 (7.1) | 1.47 (1.31-1.65) | 177 (7.9) | 1.56 (1.33-1.84) | | 200 (6.5) | | 1.38 (1.19-1.60) |
| P for interaction ^b^ | 0.090 | | | 0.032 | | 0.017 | | | |
| **Parity** |  |  |  |  |  | |  | |  |
| 1 | 7,901 (3.1) | 1,503 (5.3) | 1.36 (1.28-1.45) | 772 (5.5) | 1.31 (1.20-1.42) | | 731 (5.1) | | 1.42 (1.31-1.53) |
| 2 - 3 | 10,249 (3.7) | 1,625 (6.3) | 1.38 (1.31-1.46) | 631 (6.6) | 1.32 (1.21-1.43) | | 994 (6.2) | | 1.42 (1.33-1.52) |
| ≥4 | 2,052 (6.6) | 405 (10.8) | 1.27 (1.13-1.42) | 172 (10.7) | 1.13 (0.95-1.33) | | 233 (11.0) | | 1.38 (1.20-1.60) |
| P for interaction ^b^ | 0.392 | | | 0.234 | | 0.945 | | | |
| CI, confidence interval; CVD, cardiovascular disease; HR, hazard ratio; IR, incidence rate, per 1000 person-years; N, number; PND, perinatal depression. | | | | | | | | |  |
| ^a^ HRs were adjusted for age and calendar year (through stratifying on risk set), country of birth, marital status, education, income, smoking before pregnancy, body mass index in early pregnancy, parity, diabetes, preeclampsia, and history of psychiatric disorder, whenever applicable. | | | | | | | | | |
| ^b^ An interaction term between PND and stratification factor was added and tested for statistical significance as P for interaction. | | | | | | | |  |  |
|  | | | | | | |  | |  |

| **Supplementary Table 6.** Associations of perinatal depression with risk cardiovascular disease by time of perinatal depression diagnosis. | | | | |
| --- | --- | --- | --- | --- |
|  |  |  |  |  |
|  | **CVD** | **Model 1** | **Model 2** | **Model 3** |
|  | **N (IR)** | **HR (95% CI) ^a^** | **HR (95% CI) ^b^** | **HR (95% CI) ^c^** |
| **No PND** | 20,202 (3.6) | 1.00 | 1.00 | 1.00 |
| **Antepartum depression** | |  |  |  |
| 0-13 weeks | 927 (6.4) | 1.74 (1.63-1.86) | 1.64 (1.54-1.75) | 1.29 (1.20-1.39) |
| ≥14 weeks | 648 (6.0) | 1.64 (1.52-1.78) | 1.57 (1.45-1.70) | 1.29 (1.18-1.40) |
| **Postpartum depression** | |  |  |  |
| 0-3 months | 632 (6.0) | 1.72 (1.59-1.86) | 1.64 (1.52-1.78) | 1.38 (1.27-1.49) |
| 4-6 months | 475 (6.1) | 1.73 (1.57-1.89) | 1.63 (1.49-1.79) | 1.46 (1.33-1.61) |
| 7-12 months | 851 (5.9) | 1.68 (1.57-1.80) | 1.60 (1.49-1.71) | 1.42 (1.32-1.52) |
| CI, confidence interval; CVD, cardiovascular disease; HR, hazard ratio; IR, incidence rate, per 1000 person-years; N, number; PND, perinatal depression. | | | | |
| ^a^ HRs were adjusted for age and calendar year (through stratifying on risk set in the population-matched comparison). | | | | |
| ^b^ HRs were additionally adjusted for country of birth, marital status, education, and income. | | | | |
| ^c^ HRs were additionally adjusted for smoking before pregnancy, body mass index in early pregnancy, parity, diabetes, preeclampsia, and history of psychiatric disorder. | | | | |

| **Supplementary Table 7.** Associations of perinatal depression with risk cardiovascular disease, by time since matching. | | | | | | | | |
| --- | --- | --- | --- | --- | --- | --- | --- | --- |
|  |  |  |  |  |  |  |  |  |
|  | **No PND** | | **PND** | | **Antepartum depression** | | **Postpartum depression** | |
|  | CVD | HR | CVD | HR (95% CI) ^a^ | CVD | HR (95% CI) ^a^ | CVD | HR (95% CI) ^a^ |
|  | N (IR) |  | N (IR) |  | N (IR) |  | N (IR) |  |
| **Time since matching, years** | | |  |  |  |  |  |  |
| <1 | 1,812 (3.4) | 1.00 | 319 (5.8) | 1.33 (1.19-1.49) | 177 (7.4) | 1.09 (0.94-1.27) | 142 (4.5) | 1.78 (1.50-2.11) |
| 1-4 | 5,750 (2.7) | 1.00 | 1,047 (4.8) | 1.40 (1.31-1.49) | 428 (4.6) | 1.30 (1.17-1.44) | 619 (5.0) | 1.47 (1.35-1.59) |
| 5-9 | 7,786 (3.7) | 1.00 | 1,344 (6.2) | 1.35 (1.27-1.43) | 609 (6.4) | 1.32 (1.21-1.44) | 735 (6.2) | 1.36 (1.26-1.47) |
| ≥10 | 4,854 (5.3) | 1.00 | 823 (9.0) | 1.35 (1.25-1.46) | 361 (9.1) | 1.35 (1.20-1.51) | 462 (8.9) | 1.35 (1.22-1.48) |
| CI, confidence interval; CVD, cardiovascular disease; HR, hazard ratio; IR, incidence rate, per 1000 person-years; N, number; PND, perinatal depression. | | | | | | | | |
| ^a^ HRs were adjusted for age and calendar year (through stratifying on risk set), country of birth, marital status, education, income, smoking before pregnancy, body mass index in early pregnancy, parity, diabetes, preeclampsia, and history of psychiatric disorder. | | | | | | | | |

| **Supplementary Table 8**. Association of perinatal depression with risk of cardiovascular disease: sensitivity analyses. | | | | |
| --- | --- | --- | --- | --- |
|  |  |  |  |  |
|  | **CVD** | **Model 1** | **Model 2** | **Model 3** |
|  | **N (IR)** | **HR (95% CI) ^a^** | **HR (95% CI) ^b^** | **HR (95% CI) ^c^** |
| Clinical diagnoses of PND only | | | | |
| **No PND** | 6,380 (3.7) | 1.00 | 1.00 | 1.00 |
| **PND** | 1,104 (6.2) | 1.68 (1.58-1.79) | 1.60 (1.51-1.71) | 1.37 (1.28-1.48) |
| Antepartum | 677 (6.4) | 1.71 (1.58-1.85) | 1.63 (1.51-1.76) | 1.36 (1.23-1.49) |
| Postpartum | 427 (6.0) | 1.64 (1.49-1.80) | 1.57 (1.43-1.73) | 1.40 (1.26-1.55) |
| Primary diagnoses of CVD only | | | | |
| **No PND** | 14,695 (2.6) | 1.00 | 1.00 | 1.00 |
| **PND** | 2,330 (4.0) | 1.54 (1.47-1.60) | 1.47 (1.41-1.54) | 1.27 (1.21-1.33) |
| Antepartum | 1,041 (4.1) | 1.53 (1.44-1.63) | 1.47 (1.38-1.57) | 1.21 (1.13-1.30) |
| Postpartum | 1,289 (3.9) | 1.54 (1.45-1.63) | 1.48 (1.40-1.56) | 1.31 (1.24-1.39) |
| Excluding hypertensive disease from CVD | | | | |
| **No PND** | 13,683 (2.4) | 1.00 | 1.00 | 1.00 |
| **PND** | 2,226 (3.8) | 1.57 (1.50-1.64) | 1.51 (1.44-1.58) | 1.29 (1.22-1.35) |
| Antepartum | 987 (3.9) | 1.55 (1.45-1.65) | 1.48 (1.39-1.58) | 1.21 (1.12-1.30) |
| Postpartum | 1,239 (3.7) | 1.59 (1.50-1.68) | 1.53 (1.44-1.62) | 1.35 (1.27-1.43) |
| Excluding women with adverse pregnancy outcomes ^d^ | | | | |
| **No PND** | 10,246 (3.2) | 1.00 | 1.00 | 1.00 |
| **PND** | 2,206 (5.3) | 1.64 (1.57-1.71) | 1.56 (1.49-1.63) | 1.34 (1.28-1.41) |
| Antepartum | 954 (5.3) | 1.61 (1.51-1.72) | 1.53 (1.43-1.64) | 1.26 (1.17-1.36) |
| Postpartum | 1,252 (5.3) | 1.66 (1.57-1.76) | 1.59 (1.50-1.68) | 1.40 (1.32-1.49) |
| CI, confidence interval; CVD, cardiovascular disease; HR, hazard ratio; IR, incidence rate, per 1000 person-years; N, number; PND, perinatal depression. | | | | |
| ^a^ HRs were adjusted for age and calendar year (through stratifying on risk set in the population-matched comparison). | | | | |
| ^b^ HRs were additionally adjusted for country of birth, marital status, education, and income. | | | | |
| ^c^ HRs were additionally adjusted for smoking before pregnancy, body mass index in early pregnancy, parity, diabetes, preeclampsia, and history of psychiatric disorder. | | | | |
| ^d^ Adverse pregnancy outcomes were defined as preterm delivery, cesarean section, offspring malformation, or loss of offspring. | | | | |

**Supplementary Table 9.** Association of perinatal depression with risk of cardiovascular disease sensitivity analysis including antihypertensive medication in the definition of hypertension (women born in Sweden from 1973 onwards and giving birth between 2006-2014 were included).

|  | **No PND** | **PND** | | **Antepartum depression** | | **Postpartum depression** | |
| --- | --- | --- | --- | --- | --- | --- | --- |
|  | CVD | CVD | HR (95% CI) ^a^ | CVD | HR (95% CI) ^a^ | CVD | HR (95% CI) ^a^ |
|  | N (IR) | N (IR) |  | N (IR) |  | N (IR) |  |
| Hypertension diagnosis only | 1,435 (0.7) | 454 (1.5) | 1.65 (1.45-1.88) | 256 (1.8) | 1.56 (1.31-1.86) | 198 (1.3) | 1.74 (1.47-2.07) |
| Hypertension diagnosis + prescription of antihypertensives | 20,400 (10.6) | 6,419 (24.2) | 1.90 (1.84-1.96) | 3,165 (24.8) | 1.77 (1.69-1.85) | 3,254 (23.7) | 2.02 (1.94-2.10) |
| CI, confidence interval; CVD, cardiovascular disease; HR, hazard ratio; IR, incidence rate, per 1000 person-years; N, number; PND, perinatal depression.  ^a^ HRs were adjusted for age and calendar year (through stratifying on risk set), country of birth, marital status, education, income, smoking before pregnancy, body mass index in early pregnancy, parity, diabetes, preeclampsia, and history of psychiatric disorder, whenever applicable | | | | | | | |
